# Excess charge-carrier induced instability of hybrid perovskites

**DOI:** 10.1038/s41467-018-07438-w

**Published:** 2018-11-26

**Authors:** Yuze Lin, Bo Chen, Yanjun Fang, Jingjing Zhao, Chunxiong Bao, Zhenhua Yu, Yehao Deng, Peter N. Rudd, Yanfa Yan, Yongbo Yuan, Jinsong Huang

**Affiliations:** 1 Department of Applied Physical Sciences, University of North Carolina, Chapel Hill, NC 27599 USA; 2 Department of Mechanical and Materials Engineering, and Nebraska Center for Materials and Nanoscience, University of Nebraska-Lincoln, Lincoln, NE 68588 USA; 3 Department of Physics and Astronomy, and Wright Center for Photovoltaics Innovation and Commercialization, The University of Toledo, Toledo, OH 43606 USA; 4 Hunan Key Laboratory of Super Microstructure and Ultrafast Process, School of Physics and Electronics, Central South University, 410083 Changsha, Hunan China

## Abstract

Identifying the origin of intrinsic instability for organic–inorganic halide perovskites (OIHPs) is crucial for their application in electronic devices, including solar cells, photodetectors, radiation detectors, and light-emitting diodes, as their efficiencies or sensitivities have already been demonstrated to be competitive with commercial available devices. Here we show that free charges in OIHPs, whether generated by incident light or by current-injection from electrodes, can reduce their stability, while efficient charge extraction effectively stabilizes the perovskite materials. The excess of both holes and electrons reduce the activation energy for ion migration within OIHPs, accelerating the degradation of OIHPs, while the excess holes and electrons facilitate the migration of cations or anions, respectively. OIHP solar cells capable of efficient charge-carrier extraction show improved light stability under regular operation conditions compared to an open-circuit condition where the photo-generated charges are confined in the perovskite layers.

## Abstract (web summary)

Optoelectronic devices based on organic–inorganic halide perovskites show promising performance, but their poor stability impedes the commercialization. Here Lin et al. show that excess free charges are detrimental and efficient charge-carrier extraction is necessary for improved device stability.

## Introduction

Organic–inorganic halide perovskite (OIHP) materials have drawn tremendous attention in the field of optoelectronics^1–3^, due to their unique defect tolerance^4,5^, exceptional carrier diffusion length^6^, and many other unique properties. To date, many OIHP-based optoelectronic devices, such as solar cells^7–20^, photodetectors^21–23^, radiation detectors^24–26^, light-emitting diodes^27–29^, and others^30^, have shown even better efficiencies or sensitivities compared to their commercialized counterparts. However, device stability must be improved before OIHP optoelectronics can be a commercially viable option^31^. Stability studies on OIHP solar cells have addressed the effect of some external stimuli, such as moisture^32,33^, oxygen^34^, and ultraviolet light^35,36^. Utilizing established encapsulation techniques, the negative effects stemming from external stimuli can be reduced or avoided by providing moisture and oxygen barriers as well as protection against ultraviolet light^37^. However, encapsulated perovskite solar cells still tend to degrade under illumination, sometimes rapidly, therefore more studies are needed in order to elucidate the origin of this intrinsic degradation of perovskite materials and devices.

Here we investigate the influence of excess free charges on the stability of perovskite materials. It is found that the excess of both holes and electrons can accelerate the degradation of perovskite materials, which can be explained by the excess free charges facilitating ion migration within OIHPs. The photo-generated electrons and holes show different impacts on the migration of cations and anions, with reduced migration energy barriers for cation by holes and for anion by electrons. Finally, the excess-charge induced material degradation is shown to be suppressed by efficient extraction of the photo-generated charges in efficient solar cells.

## Results

### Excess charge induced phase separation of perovskites

**Fig. 1:**
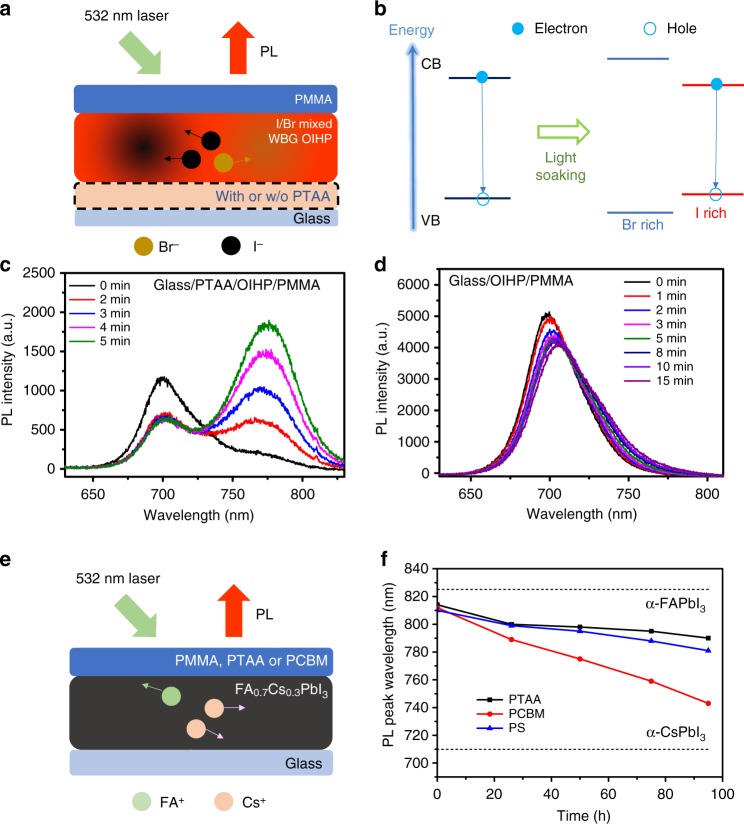
Excess charge induced phase separation of OIHPs. **a** A scheme of PL measurement setup for FA_0.85_Cs_0.15_Pb(I_0.6_Br_0.4_)_3_ with and without PTAA layer. **b** A scheme of light induced PL red-shift within mixed Br/I OIHPs. **c** PL of glass/PTAA/FA_0.85_Cs_0.15_Pb(I_0.6_Br_0.4_)_3_/PMMA. **d** PL of glass/FA_0.85_Cs_0.15_Pb(I_0.6_Br_0.4_)_3_/PMMA. **e** A scheme of PL measurement setup for mixed cation FA_0.7_Cs_0.3_PbI_3_ covered by PTAA, PCBM or PMMA. **f** The relationship of PL peak wavelength versus illuminated time of FA_0.7_Cs_0.3_PbI_3_ films with different covers

The excess charge induced instability was discovered while investigating the light stability of wide-bandgap (WBG) OIHPs for application in OIHP/silicon tandem solar cells. Though it has been well established that some mixed halide perovskites have phase separation under illumination^38–43^, an anomalously fast phase separation of a WBG OIHP, FA_0.85_Cs_0.15_Pb(I_0.6_Br_0.4_)_3_ (FA = HC(NH_2_)_2_), was observed under illumination when deposited on poly(triaryl amine) (PTAA) coated glass substrates, as compared to control samples deposited directly on glass substrate. Schemes of the measurement setup and effect of phase separation in mixed halide perovskites can be seen in Fig. 1a, b, respectively. As shown in Fig. 1c, the FA_0.85_Cs_0.15_Pb(I_0.6_Br_0.4_)_3_ film on PTAA showed a new red-shifted photoluminescence (PL) peak, ca. 760 nm in comparison with its main PL peak at 700 nm after illumination for 1–2 min by laser light (532 nm, 100 mW cm^-2^), which represents a typical light-induced phase separation in mixed halide perovskites^38^. The resulting intensity of the 760 nm PL peak showed a four folds increase after illumination for 5 min. In striking contrast, perovskite film formed from the same precursor solution on glass substrate showed negligible phase separation, lacking the red-shifted PL peak even after illumination for 15 min (Fig. 1d).

In order to clarify the origin of the accelerated phase separation in FA_0.85_Cs_0.15_Pb(I_0.6_Br_0.4_)_3_ on PTAA, we first checked for possible differences in composition and grain size of the WBG OIHP, as varied composition or grain size has been shown to result in different phase-separation dynamics^44^. Different substrates may influence the process of OIHP grain formation inducing variations in grain size and composition^45^. The FA_0.85_Cs_0.15_Pb(I_0.6_Br_0.4_)_3_ films on both PTAA-substrate and glass-substrate showed the PL peaks at *ca*. 700 nm, indicative of both films sharing a similar composition. The grain sizes, and size distribution, of the FA_0.85_Cs_0.15_Pb(I_0.6_Br_0.4_)_3_ films on glass/PTAA- and glass-substrate measured by scanning electron microscopy (SEM) (Supplementary Figure 1) were found to be very similar. One test that can exclude differences in film composition or morphology as a cause for the observed phenomenon is to cover the FA_0.85_Cs_0.15_Pb(I_0.6_Br_0.4_)_3_ films, deposited from the same solution on glass, with PTAA or insulating poly(methyl methacrylate) (PMMA). The sample covered by PTAA still showed much faster phase separation (Supplementary Figure 2) than that covered by PMMA, confirming that the presence of a PTAA layer accelerates phase separation in FA_0.85_Cs_0.15_Pb(I_0.6_Br_0.4_)_3_ under illumination.

One crucial difference between PTAA and PMMA is that PTAA is a hole transport layer (HTL) material which can extract photo-generated holes and leave a large density of excess electrons in the OIHP layer^46^. Thus, we speculate that the resulting excess of electrons accelerate the phase separation of FA_0.85_Cs_0.15_Pb(I_0.6_Br_0.4_)_3_. This speculation was verified by showing that the extraction of photo-generated electrons by an electron transporting layer (ETL) of [6]-phenyl-C61-butyric acid methyl ester (PCBM) was capable of mitigating the PL peak shift associated with phase separation, even after illuminating the PCBM covered FA_0.85_Cs_0.15_Pb(I_0.6_Br_0.4_)_3_ film for 15 min with the same light source (Supplementary Figure 2), while PL intensity of the perovskite covered by PCBM increased. Furthermore, we investigated light induced PL evolution of FA_0.85_Cs_0.15_Pb(I_0.6_Br_0.4_)_3_ films with different covering layers in N_2_ environment to exclude the possible impact of oxygen or moisture to the measurement. As shown in Supplementary Figure 3, PTAA covered FA_0.85_Cs_0.15_Pb(I_0.6_Br_0.4_)_3_ films also showed fast phase separation. In contrast, PMMA and PCBM-covered films did not. Since the phase separation of WBG OIHP has been established to be induced by halide migration under illumination^38,47^, the unextracted excess electrons in PTAA-covered FA_0.85_Cs_0.15_Pb(I_0.6_Br_0.4_)_3_ should play a critical role in promoting the migration of halide anions.

The absence of PL peak shift for PCBM-covered FA_0.85_Cs_0.15_Pb(I_0.6_Br_0.4_) also indicates that resulting excess holes do not obviously influence the migration of the halide anions. However, it cannot provide information on influence of excess holes on the ion migration of cations in OIHP. To find it out, we investigated the PL variation of iodinated OIHP with mixed cations, FA_0.7_Cs_0.3_PbI_3_, under illumination with the perovskite films covered by PTAA, PCBM, or insulating polystyrene (PS) (Fig. 1e). Not iodine migration but cation migration may induce local composition transformation, and then potential PL shift of FA_0.7_Cs_0.3_PbI_3_ under illumination. Before illumination, all of the fresh FA_0.7_Cs_0.3_PbI_3_ films exhibited a PL peak at *ca*. 815 nm (Supplementary Figure 4). After illumination under white light with intensity of 100 mW cm^-2^ for 26 h, all the three FA_0.7_Cs_0.3_PbI_3_ films covered by PTAA, PCBM and PS showed a blue-shift in PL (Supplementary Figure 4). The blue-shifting of PL peaks may be attributed to the formation of a Cs-rich phase in OIHP resulting from FA/Cs migration, because the PL peak of α-CsPbI_3_ (710 nm)^48^ is observed at a shorter wavelength than α-FAPbI_3_ (825 nm)^49^. One possible explanation for the blue shift of PL observed for the FA/Cs mixed perovskites after illumination is that the FA-rich phase may not form the α-phase perovskite which has a lower bandgap than the matrix. The FA-rich black phase perovskite is thermodynamically unstable at testing temperature, and thus yellow color β-phase perovskite most likely will form. Another possible reason is that FA migrate to surface of samples and then volatilize like MA, thus the remaining perovskite film is Cs-rich composition, which has a larger bandgap. We did not observe the PL recovery of FA_0.7_Cs_0.3_PbI_3_ in the dark in several days, which can be attributed to β-phase FA-rich perovskite or FA volatilization. We can not exclude the possibility that the cation back diffusion in the illuminated-FA_0.7_Cs_0.3_PbI_3_ in the dark is too slow to be observed. After illumination for 75 h, the PCBM-covered film exhibited the greatest blue shift in its PL resulting in a 55 nm shift to *ca*. 760 nm (Supplementary Figure 4). In contrast, the FA_0.7_Cs_0.3_PbI_3_ covered by PTAA and PS layers showed obviously smaller shifts in PL of 19 nm and 25 nm, respectively. Figure 1f summarizes the time-dependent PL peak position versus illumination time of FA_0.7_Cs_0.3_PbI_3_ films with different covering layers, in which PCBM covered FA_0.7_Cs_0.3_PbI_3_ showed the most rapid shift in PL, which can highly possible be attributed to the light-induced local composition transformation via cation migration. Thus, the unextracted excess holes in ETL-covered OIHP may facilitate the cation migration in OIHPs.

### Impact of excess free charges on ion migration

**Fig. 2:**
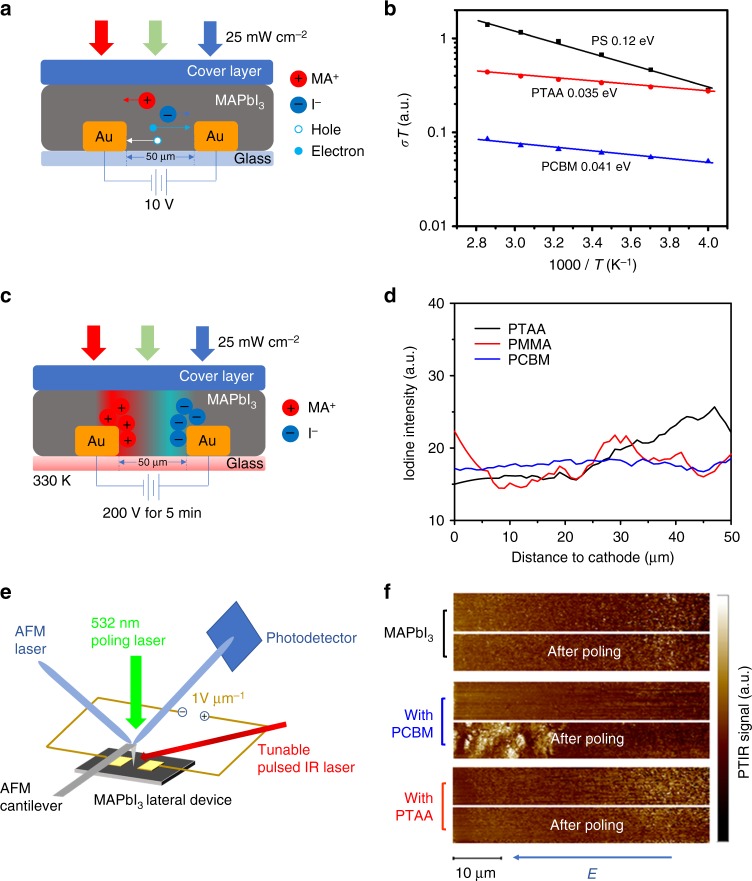
Facilitation of ion migration within OIHP films by excess free charges. **a** A scheme of the conductivity measurement setup for OIHP covered by HTL, ETL, and insulated layer under illumination at light intensity of 25 mW cm^-2^. **b** The temperature-dependent conductivity of MAPbI_3_ covered by PTAA, PCBM, and PS. **c** A scheme of setup of poling OIHP covered by PTAA, PCBM and PMMA. **d** EDS of MAPbI_3_ films covered by PTAA, PMMA, and PCBM after poling under illumination, the noise level of intensity is *ca*. ±1 (a.u.). **e** A scheme of PTIR measurement setup. **f** PTIR mapping of MAPbI_3_ film as well as those covered by PCBM and PTAA, at the same location before and after poling under illumination

The PL study indicates that excess electrons and holes can promote the anion or cation migration in the two perovskites with mixed anions and cations, respectively. We speculate that the influence of excess charges to ion migration should be general to all OIHPs. To confirm this, we measured the activation energy (*E*_a_) for ion migration of OIHPs under illumination covered by HTL, ETL, and insulating layer. The ionic conductivity in a solid is determined by the *E*_a_ utilizing the Nernst–Einstein relation (1):

1 $$\sigma (T) = \frac{{\sigma _0}}{T}\exp \left( {\frac{{ - E_{\mathrm{a}}}}{{kT}}} \right),$$

where *σ*_0_ is a constant, *k* is the Boltzmann constant, *T* is the absolute temperature, and *E*_a_ can be derived from the slope of the ln(*σT*)-1/*kT* relation. The activation energy of ion migration directly characterizes how easily ions are able to move^50,51^. The *E*_a_ of ion migration was thus extracted from the temperature dependent conductivity of the OIHP films in lateral-structure devices^52^ consisting of two gold (Au) electrodes deposited on OIHP polycrystalline films^53,54^. Here CH_3_NH_3_PbI_3_ (MAPbI_3_) was chosen as the perovskite layer which was covered by PTAA, PCBM, PS or PMMA layers, as illustrated in Fig. 2a, and bottom electrodes only contacted with perovskites to exclude current signal from cover layers. The polycrystalline MAPbI_3_ films were deposited by the one-step anti-solvent method which can produce high performance solar cells with power conversion efficiency (PCE) up to 20.5%^14^. In this measurement, a small constant electric field of 0.2 V μm^-1^ was applied, which was set to be comparable to the operating condition in the solar cells. Previous studies already established that a transition from electronic conduction to ionic conduction was generally observed at elevated temperature in three-dimensional perovskites, and the faster change of film conductivity at high temperatures is caused by ion migration^53^, and the *E*_a_ for ion migration was calculated from the slope of the ln(*σT*) versus 1/*kT* relation at high temperature region of 1000/*T* less than *ca*. 4 K^-1^. The ion conduction activation energy in polycrystalline MAPbI_3_ film with grain size of *ca*. 1 μm generally reduces from *ca*. 0.50 eV in the dark to *ca*. 0.14 eV under illumination by white light at an intensity of 25 mW cm^-2^^53^. Here we observed that both HTL-covered and ETL-covered MAPbI_3_ films showed much smaller *E*_A_ of *ca*. 0.04 eV than the MAPbI_3_ films covered by PS or PMMA (Fig. 2b and Supplementary Figure 5). It should be noted that such a small activation energy is already comparable to the de-trapping energy of trapped charge carriers. This quantitatively proves that ion migration is much easier in the OIHP films with excess holes or electrons.

The effect of excess charges on anion and cation migration in OIHP was further visualized at the microscopic level using energy-dispersive X-ray spectroscopy (EDS) and photothermal induced resonance (PTIR) microscopy to detect the I^−^ and MA^+^ redistribution in MAPbI_3_ films during electrical poling. For EDS measurement, lateral-structure devices shown in Fig. 2c were used. EDS line scanning was carried out to trace the iodide anion redistribution due to their poling induced migration. As shown in Fig. 2d, after poling under a field of 4 V μm^−1^ for 300 s at 330 K and illumination with white light, a clear iodine concentration gradient was observed with iodine concentration increasing from the cathode to the anode for the PTAA covered MAPbI_3_ film, while the iodine concentration in PCBM covered film remained uniform between two electrodes after same poling and illumination process. The PMMA covered film showed a weaker iodine migration than PTAA-covered MAPbI_3_ with a smaller concentration gradient. These results provide direct evidence that the excess electrons can increase the anion migration in polycrystalline MAPbI_3_ thin films, while extracting excess electrons suppresses the migration of I^−^ anions.

Since EDS has little sensitivity to the MA^+^ cation, we applied PTIR microscopy to map the redistribution of organic cations during the poling process. MA^+^ has strong infrared (IR) absorption at *ca*. 1460 cm^−1^, which induces a larger local thermal expansion of the film when the film is excited by IR laser with wavelength tuned to match the absorption of MA^+^. The variation of local thermal expansion of the film can thus reflect the relative concentration distribution of MA^+^^54,55^. The lateral resolution of PTIR is ~30 nm. The PTIR measurement setup is shown in Fig. 2e, and the PTIR images for MA^+^ distribution obtained before and right after poling are shown in Fig. 2f. After poling under an electric field of 1 V µm^−1^ and illuminating for 60 s (30 mW cm^−2^, 532 nm), the PCBM-covered MAPbI_3_ showed a distinct increase in MA^+^ content at the cathode side, which resulted from MA^+^ migration from anode to cathode. In contrast, the PTIR images of the other two MAPbI_3_ films show no obvious MA^+^ redistribution after poling. The bright lines along the Au electrode edge are caused by plasmonic effect of the Au electrodes which enhances PTIR signal intensity. These results provide direct evidence for the excess of holes in MAPbI_3_ accelerating the migration of MA^+^ cations.

### Impact of excess free charge on perovskite stability

**Fig. 3:**
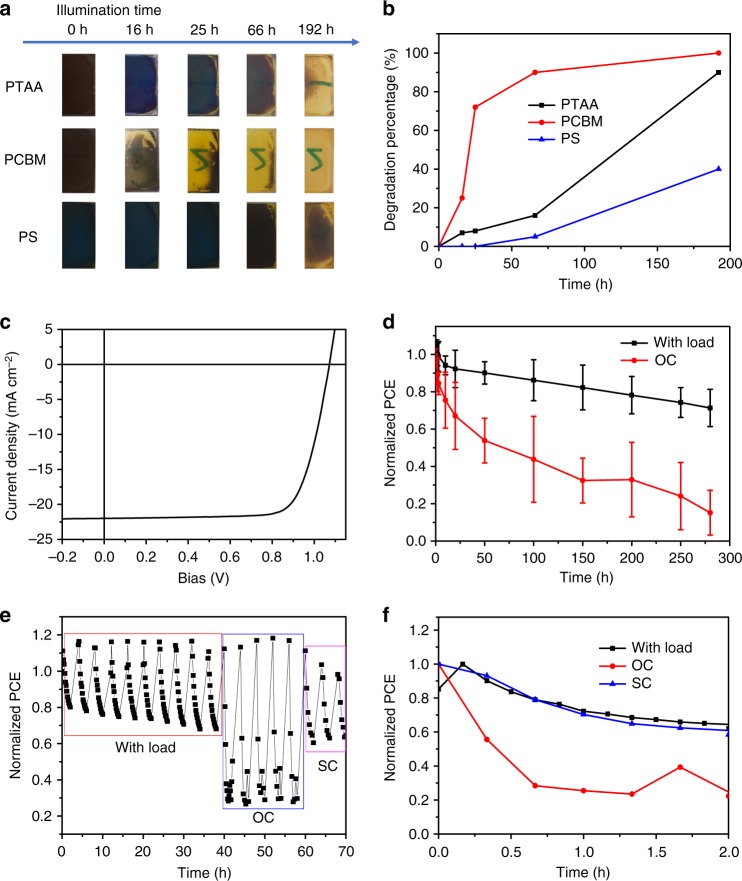
Impact of excess free charge on stability of OIHP thin films and solar cells under illumination. **a** Photographs and **b** degradation percentage of the MAPbI_3_ films covered by PTAA, PCBM, and PS under illumination for different time. **c** The typical original *J–V* curve of devices for stability test. **d** The light stability of devices with load and in open circuit (OC) condition under continuous illumination, the error bar (±standard deviation) were calculated from 5 devices. **e**, **f** The typical light stability of degraded device at different conditions: with load, OC and short circuit (SC), under light and dark cycling

We studied the long-term light stability of MAPbI_3_ thin films covered by PTAA, PCBM and PS to examine the influence of excess charge carriers on the stability of perovskite films. It is established that MAPbI_3_ can decompose to PbI_2_, MA^+^ and I^−^ under illumination, resulting in discoloration of the films^33^, and both MA^+^ and I^−^ migration can accelerate this chemical decomposition. The photographs (Fig. 3a) and degradation percentage (Fig. 3b) for different illumination times showed that PTAA- and PCBM-covered MAPbI_3_ films degraded faster than the PS-covered film, which can be attributed to the facilitation of I^−^ or MA^+^ migration by excess electrons or holes, respectively. These results confirm that both excess electrons and holes can accelerate the degradation of perovskite materials. Thus, efficient charge extraction is necessary for light stable OIHP materials.

To study the influence of excess charge carriers on the stability of OIHP solar cells, the light stability of MAPbI_3_ solar cells with and without loads were compared. The load resistance was chosen so that the device operated at the maximum power point at the beginning of the test, the load was then fixed for the duration of the measurement. The device has a *p-i-n* planar structure: indium tin oxide (ITO)/PTAA/MAPbI_3_/PCBM/C60/bathocuproine (BCP)/cupper (Cu), and we encapsulated device to eliminate the effect of external factors, such as moisture and oxygen. Figure 3c showed the typical *J*–*V* curve of the devices which were selected to study light stability. The solar cells with loads exhibited an evident improvement in light stability compared to devices in open-circuit conditions (Fig. 3d). After continuous illumination at 100 mW cm^-2^ for 280 h (Fig. 3d), the PCEs of the devices in open-circuit condition showed *ca*. 85% decrease on average, in contrast the devices with loads showed a degradation of *ca*. 30% on average after illumination for 280 h. To further confirm this phenomenon, we measured the light stability of OIHP devices which have been pre-illuminated under 100 mW cm^-2^ white light at 333 K for 500 h. These pre-degraded devices have more defects and mobile ions than the fresh devices, where unextracted charge carriers can more easily accelerate device degradation. We measure the stability of the same device under different conditions, open-circuit, short-circuit, and with load. After cycles of testing under light and in the dark for 2 h each cycle, the device performance can mostly be recovered. As shown in Fig. 3e, f, the device performance at short-circuit condition showed similar degradation to that with load. In striking contrast, there was a drastic decrease in device PCE after illumination under open-circuit conditions. This phenomenon had proved to have very good reproducibility if the starting cells were fabricated under similar conditions. This result again verifies that the excess-charge induced instability of OIHP solar cells can be suppressed through efficient charge extraction.

## Discussion

**Fig. 4:**
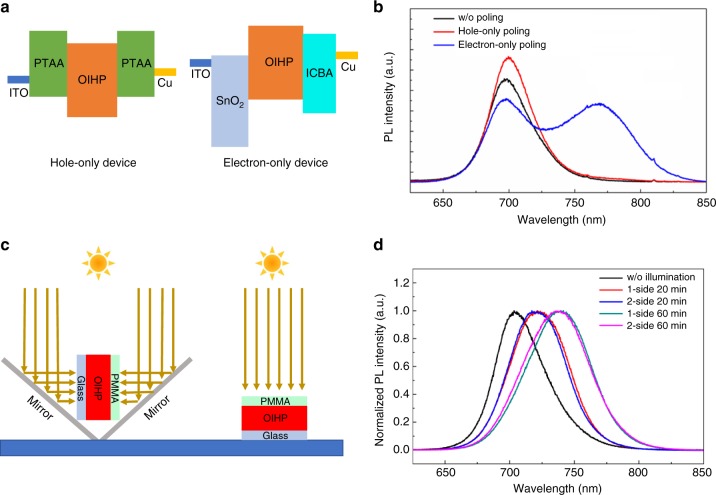
Impact of excess free charges and their distribution on stability of OIHP. **a** Schematic structure of hole-only and electron-only device, and OIHP is FA_0.85_Cs_0.15_Pb(I_0.6_Br_0.4_)_3_. **b** PL spectra evolution of hole-only and electron only devices under electric poling. **c** A scheme of PL measurement setup under illumination from PMMA side or both glass and PTAA sides. **d** PL spectra evolution of glass/OIHP/PMMA under single side or double side illumination at 100 mW cm^-2^ white light

To ascertain the origin of light induced instability of OIHP materials, we first examined whether it is free charges generated by incident light that caused the material instability under illumination. Here we injected free charges by an applied electric field into mixed halide OIHP, FA_0.85_Cs_0.15_Pb(I_0.6_Br_0.4_)_3_, and investigated the resulting phase separation behavior. To separate the function of free holes and electrons, we fabricated hole-only and electron-only devices with a vertical structure of ITO/PTAA/OIHP/PTAA/Cu or ITO/ tin oxide (SnO_2_)/OIHP/indene-C_60_ bisadduct (ICBA)/Cu, respectively (Fig. 4a). The PL of FA_0.85_Cs_0.15_Pb(I_0.6_Br_0.4_)_3_ was recorded after electrical poling in the dark. A new PL peak appeared at 770 nm after poling the SnO_2_/OIHP/ICBA electron-only device at 3 V µm^−1^ for 60 min (Fig. 4b), in sharp contrast, we did not observe a shift in the PL peak after poling the PTAA/OIHP/PTAA hole-only device at 7 V µm^−1^ with the same poling time but higher current density than electron-only device. These results indicate that the illumination induced OIHP instability is caused by the excess photo-generated charge carriers promoting ion migration within OIHPs. In addition, it also confirms that halide phase separation in FA_0.85_Cs_0.15_Pb(I_0.6_Br_0.4_)_3_ is caused by excess electrons, rather than excess holes.

The final question left to be addressed is how the excess electrons (holes) enhance the phase separation of anions (cations) or ion migration rate in OIHP. Current theoretical studies predict that ion migration in OIHP polycrystalline films mainly occur through their structural defects, either point or extended defects^56–59^. Thus, the results reported here show that both excess holes and electrons can decrease the energy barrier for ion migration, which should have an effect on defect formation in perovskite materials. We considered several possible mechanisms that may explain the observed phenomenon: (1) The non-uniform distribution of photo-generated charges imposes an electric field which can induce a strain in the material by electromechanical effects (including piezoelectric and electrostriction effects)^60^. We already demonstrated that an applied field can add strain to the perovskites which reduces the energy barrier for ion migration and thus accelerating material degradation^60^; (2) charge distribution induced electric field may weaken interionic interaction, and then facilitate the generation of more structural defects, which can promote ion migration in polycrystalline OIHP predicted by current theoretical studie^56–59^; (3) excess free charges screen and weaken interionic interaction, such as ionic bonding and hydrogen bonding, within OIHP lattices, which leads to easier ion migration; (4) the excess charges neutralize the ion vacancies generated by leaving ions, thus reducing the drag force induced by the attractive columbic force between the vacancy and migrant ion. Since both scenarios (1) and (2) are related to the electric field, here we compared PL spectrum evolution of mixed halide FA_0.85_Cs_0.15_Pb(I_0.6_Br_0.4_)_3_ (its optical density seen in Supplementary Figure 6) with light illumination coming from one side and both sides in order to simulate two cases with and without internal field induced by the charge distribution (Fig. 4c). Nevertheless, we do not see obvious difference in PL variation trend or rate (Fig. 4d), which excludes the non-uniform charge distribution as an origin of the accelerated phase separation in OIHPs. It should be noted that a similar study done by Barker et al.^39^ showed different results with our findings here, indicating that further work is still required to understand the difference of methods used. One difference is that we used white light in this study to excite perovskite films where light with different wavelength may cause different distribution of generated charges. Particularly the long wavelength light may cause a non-exponential charge generation rate curve, but their contribution to charges is also small due to the weaker absorption. Additional evidence that rules out the contribution of electric field to the faster ion migration is the different phase separation behavior observed in the electron-only and hole-only devices shown in Fig. 4b where both devices were under electric field.

**Fig. 5:**
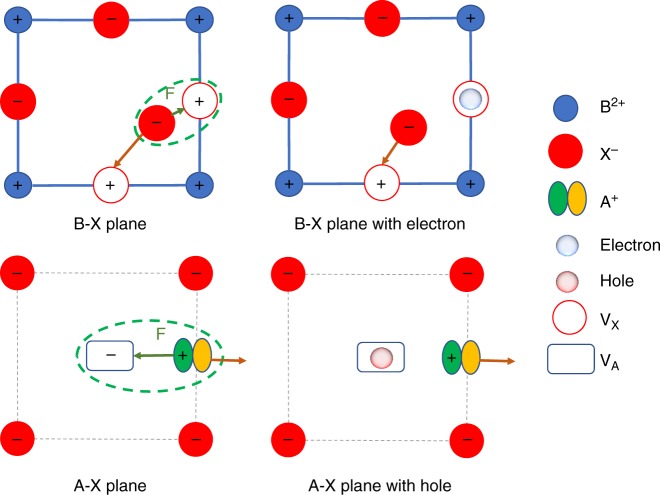
Proposed mechanism for charge induced instability of OIHP. Charges neutralize the vacancy generated by leaving the ions and then reduce ion migration dragging force which is the coulombic attraction induced by the vacancies and moving ions. This applies for both type of charges. In B–X plane, the V_x_ is neutralized by the excess electron, and then X^-^ migration is promoted; and in A–X plane, the V_A_ is neutralized by the excess hole, and then A^+^ migration is promoted

The charge carrier concentration (*ca*. 10^15^ cm^-3^) under illumination of 25 mW cm^-2^ is much lower than the ion density (*ca*. 10^22^ cm^-3^) in ABX_3_ perovskite, therefore it is unlikely that the very low concentration free charges could change the lattice constant of the perovskite. We thus believe the last mechanism is more reasonable which is illustrated in Fig. 5. For ionic materials such as halide perovskites, ion migration has additional energy barrier coming from the coulombic attraction induced by the ion vacancies and moving ions. The excess charges that refer to the photo-generated carriers or carriers injected by current, can neutralize the ion vacancies and then reduce dragging force associated with ion migration, leading to increased ion migration and reduced stability of OIHPs. For halide migration, the X vacancy (V_x_), which is positively charged, is generated by the leaving X^−^ ion, like I^−^, from the X site of an ABX_3_ lattice. Compared to the sites without defects, electrons prefer to localize in V_x_ and neutralize them due to the coulombic attraction; then migration of X^−^ is promoted due to the weakened or annulled attraction between the V_x_ and migrating X^−^. Similarly, for A site cation migration, the A site vacancies (V_A_) with negative charges can be neutralized by the excess holes, thus facilitating the migration of A^+^ cations. Since halide perovskites are mostly intrinsic or weakly *p*-type, the free charge density in perovskites is small, and thus excess charges are needed in accelerating the ion migration. It should be noted that our study mainly explains how excess free charges change ion migration rate, however, the driven force for phase segregation in the mixed cation and anion perovskites under illumination remains to be an open question^40,42^.

In conclusion, through experimental analysis, excess charge carriers were found to accelerate the ion migration in OIHP materials, which could be one of the major limitations for the light stability of OIHP thin films and perovskite optoelectronic devices. We verified that both holes and electrons could decrease *E*_a_ of ion migration within OIHP films, and holes and electrons mainly boost cation or anion migration, respectively. This discovery is crucial for understanding the role of excess charge carriers in stability of OIHP based optoelectronic devices. Efficient charge extraction from OIHPs has been shown to suppress ion migration and device degradation in OIHP solar cells under light, which can be broadly applicable to many other OIHP optoelectronic applications, such as photodetectors and radiation detectors.

## Methods

### Fabrication of OIHP thin-film and lateral devices

The spin coating process was conducted in glovebox with oxygen level lower than 100 parts per million. All OIHP thin films were deposited by using anti-solvent method. The perovskite precursor solution composed of mixed cations and halides was dissolved in mixed solvent of *N*, *N*-dimethylformamide (DMF): dimethyl sulfoxide (DMSO). Unless stated otherwise, the volume ratio of DMF and DMSO is 9:1 for MAPbI_3_ and 4:1 for the other composition. Then 80 μL precursor solution was spun onto substrate at 2000 rpm for 2 s and 4000 rpm for 20 s, the sample was quickly washed with 130 μL toluene during the 4000 rpm spin-coating. Subsequently, the sample was annealed at 65 °C for 10 min and 100 °C for 10 min. The thickness of OIHP is *ca*. 500 nm, which was measured using a Bruker Dektak-XT stylus profilometer. The 50 nm Au electrodes (spaced by 50 μm) for lateral devices were thermally deposited. The PTAA was dissolved in toluene, and PCBM, PMMA and PS were dissolved in 1,2-dicholorobenze, and then spin-coated on the OIHP thin films. Finally, the samples were thermally annealed at 100 °C for 10 min.

### Ion migration activation energy characterization

The measurements were performed in a Lakeshore Probe Station with white light through a quartz window. The samples were placed on a metal plate with its temperature being controlled by a heater and injected liquid N_2_. For each conductivity, the current through the devices were stabilized for 5 min when an objective temperature was reached, before the current measurement was performed. A semiconductor analyzer (Keithley 2400) was used for the current measurement with an applied bias of 10 V.

### Optical and structure characterization

The PL spectrum was measured from OIHP topsides by iHR320 Photoluminescence Spectroscopy at room temperature. A 532-nm green laser from Laserglow Technologies was used as the excitation source in PL measurement. PL was measured in air, unless stated otherwise. For PL study of mixed halide perovskites illuminated from one/both sides, we illuminated from one/both sides to boost halide migration by using white light with intensity of 86.5 mW cm^-2^, and then immediately measured them PL peak to see phase separation using 532-nm laser for excitation. Top view scanning electron microscope (SEM) images were obtained with a Quanta 200 FEG ESEM. The UV–vis absorption spectrum of perovskite thin film was recorded using a Thermo Scientific Evolution 201 UV–Visible spectrophotometer.

### PTIR characterization

PTIR experiments were carried out in air using a nanoIR2 system (ANASYS Instruments) that consists of an atomic force microscope (AFM) microscope operating in contact mode and a tunable pulsed laser source. The laser emits pulses about 300 ns long that are tunable from 1965 to 910 cm^−1^. The laser illumination was from the topside of the sample and the typical laser spot size was around 30 μm on the sample. PTIR spectra were obtained by averaging the cantilever deflection amplitude from 128 individual laser pulses at each wavelength and tuning the laser at intervals of 2 cm^−1^. PTIR images were recorded by illuminating the sample with a constant wavenumber of 1460 cm^-1^ while scanning with the AFM tip.

### EDS measurement

EDS was carried out on a FEI Helios NanoLab TM 660 instrument equipped with TEAM energy dispersive spectroscope. The typical electron acceleration voltage and current for X-ray excitation was 15 kV and 100 pA. Line scan resolution is 1 μm, 8 frames with dwell time of 500 ms. The MAPbI_3_ thin film for EDS measurement was deposited from the precursor solution with pure DMF solvent.

### Fabrication and stability test of solar cells

The solar cell device structure was ITO/PTAA/MAPbI_3_/PCBM/C60/BCP/Cu. The spin-coating process was conducted in a glovebox with oxygen level lower than 100 parts per million. The PTAA was dissolved in toluene with a concentration of 2 mg mL^-1^, and spin-coated on the ITO/glass substrates at the speed of 5000 rpm. The spun PTAA films were thermally annealed at 100 °C for 10 min. MAPbI_3_ film was deposited by using the same method we mentioned above in the lateral device fabrication. After annealing, PCBM were deposited on top of MAPbI_3_ by solution spin-coating from *o*-dichlorobenzene solution (20 mg mL^-1^) at a speed of 6000 rpm, and then annealing at 100 °C for 30 min. After the deposition of PCBM layer, 20 nm thickness of C60 was thermally evaporated with a deposition rate of 0.5 Å s^-1^. The devices were finished by the evaporation of 8 nm BCP and 80 nm Cu electrode. The device area is 8 mm^2^. The devices were encapsulated by using epoxy with glass slides. For stability test, the light was from a 300 W plasma lamp and the intensity on the devices was about 100 mW cm^-2^. The *J–V* scan direction is from positive bias to negative bias, and *J–V* curves were corrected using a Keithley 2400 source meter.

## Electronic supplementary material

Supplementary Information

## Data Availability

The data that support the findings of this study are available from the corresponding author on reasonable request.
