# Supplementary material for: Excess charge-carrier induced instability of hybrid perovskites
Source: Nat Commun. 2018 Nov 26;9:4981. doi: 10.1038/s41467-018-07438-w (PMC6255755; doi:10.1038/s41467-018-07438-w)
Supplement: Supplementary file 1 — Supplementary Information [file 41467_2018_7438_MOESM1_ESM.pdf]

Supporting information for

Excess Charge-Carrier Induced Instability of Hybrid Perovskites

Yuze Lin[1], Bo Chen[2], Yanjun Fang[2], Jingjing Zhao[1,2], Chunxiong Bao[2], Zhenhua Yu[1], Yehao Deng[1], Peter. N. Rudd[1], Yanfa Yan[3], Yongbo Yuan[4], and Jinsong Huang[1,2]*

[1]Department of Applied Physical Sciences, University of North Carolina, Chapel Hill, NC 27599, USA.

[2]Department of Mechanical and Materials Engineering and Nebraska Center for Materials and Nanoscience, University of Nebraska-Lincoln, Lincoln, NE 68588, USA.

[3]Department of Physics and Astronomy, and Wright Center for Photovoltaics Innovation and Commercialization, University of Toledo, Toledo, Ohio 43606, USA.

[4]Hunan Key Laboratory of Super Microstructure and Ultrafast Process, School of Physics and Electronics, Central South University, Changsha, Hunan 410083, P. R. China.

[*] Correspondence to JH, Email: jhuang@unc.edu

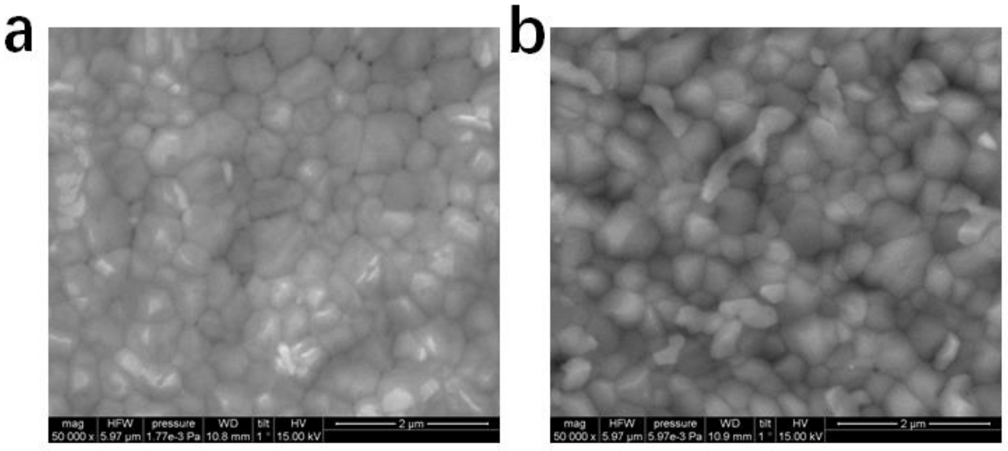

Supplementary Figure 1 SEM morphology of WBG OIHP. Top view SEM images of $FA_{0.85}Cs_{0.15}Pb(I_{0.6}Br_{0.4})_3$ films on (a) glass/PTAA- and (b) glass-substrates.

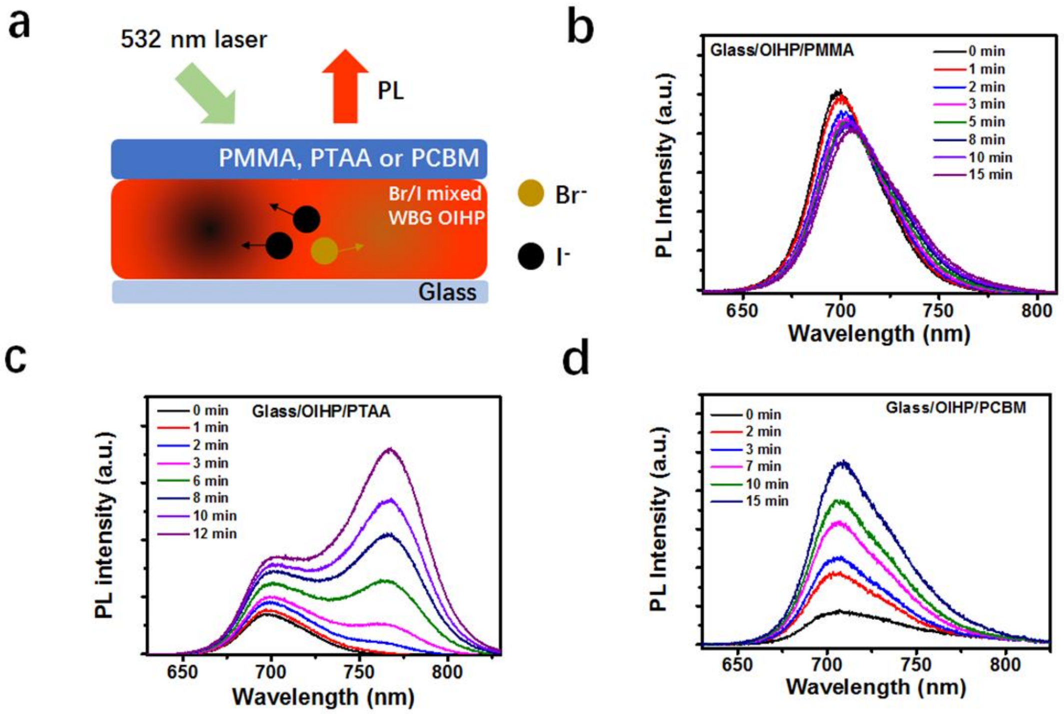

Supplementary Figure 2 Phase separation of WBG OIHP with different covers. (a) A scheme of PL measurement setup for $FA_{0.85}Cs_{0.15}Pb(I_{0.6}Br_{0.4})_3$ covered by PTAA, PCBM or PMMA. PL of glass/$FA_{0.85}Cs_{0.15}Pb(I_{0.6}Br_{0.4})_3$ covered by (b) PMMA, (c) PTAA and (d) PCBM after illumination.

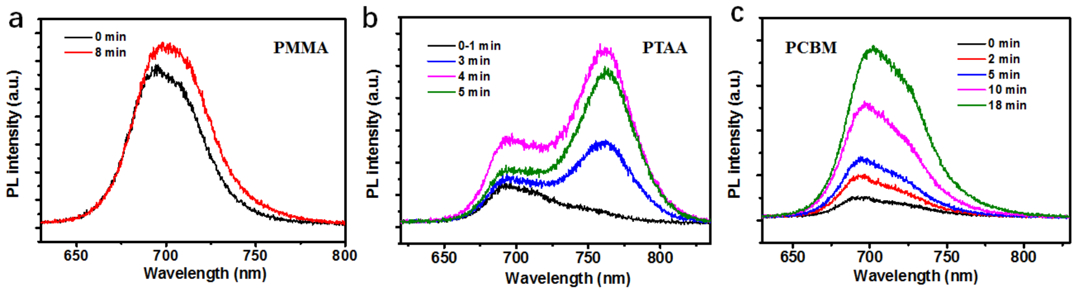

Supplementary Figure 3 Phase separation of WBG OIHP with different covers in N_2. PL of glass/$FA_{0.85}Cs_{0.15}Pb(I_{0.6}Br_{0.4})_3$ covered by (a) PMMA, (b) PTAA and (c) PCBM after illumination in N_2.

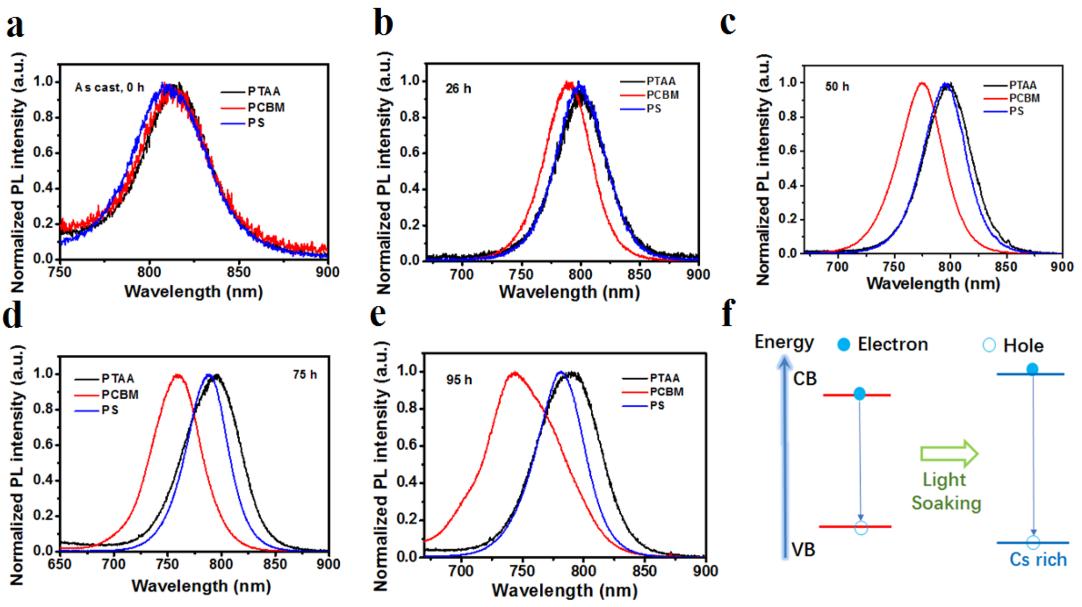

Supplementary Figure 4 Phase separation of mixed cation OIHP with different covers. PL of glass/$FA_{0.7}Cs_{0.3}PbI_3$ covered by PTAA, PCBM and PS after illumination 100 mW cm^{-2} white light for (a) 0 h, (b) 26 h, (c) 50 h, (d) 75 h and (e) 95 h. (f) A proposed mechanism of light induced PL blue shift of $FA_{0.7}Cs_{0.3}PbI_3$.

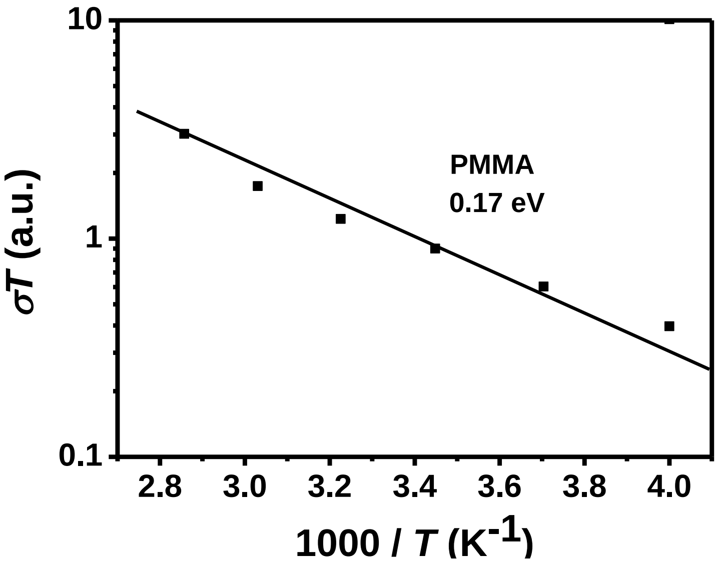

Supplementary Figure 5 Active energy of ion migration of $MAPbI_3$ covered by PMMA. The temperature-dependent conductivity of $MAPbI_3$ covered by PMMA under illumination.

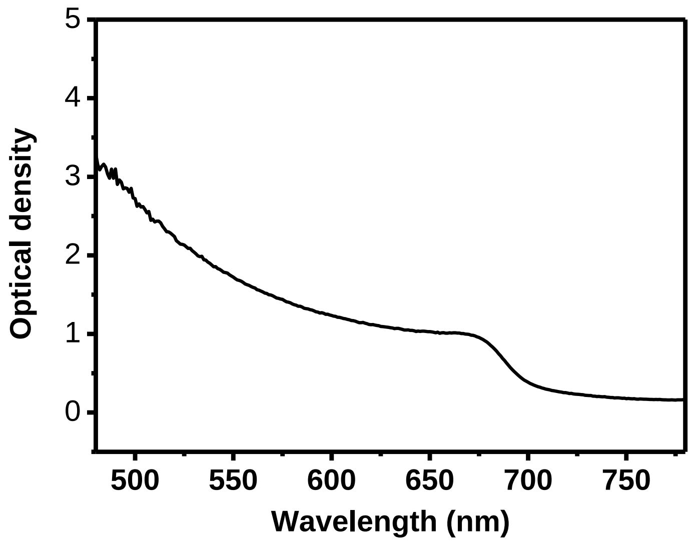

Supplementary Figure 6 Absorption of wide bandgap perovskite. Absorption spectrum of $FA_{0.85}Cs_{0.15}Pb(I_{0.6}Br_{0.4})_3$ on glass substrate.
